# Metformin and salinomycin as the best combination for the eradication of NSCLC monolayer cells and their alveospheres (cancer stem cells) irrespective of EGFR, KRAS, EML4/ALK and LKB1 status

**DOI:** 10.18632/oncotarget.2657

**Published:** 2014-11-02

**Authors:** Zhiguang Xiao, Bianca Sperl, Axel Ullrich, Pjotr Knyazev

**Affiliations:** ^1^ Department of Molecular Biology, Max-Planck-Institute of Biochemistry, Am Klopferspitz, Martinsried, Germany

**Keywords:** NSCLC, CSCs, combinatorial treatment, Metformin, Salinomycin

## Abstract

The presence of cancer stem cells (CSCs) is linked to preexisting or acquired drug resistance and tumor relapse. Therefore, targeting both differentiated tumor cells and CSCs was suggested as an effective approach for non-small cell lung cancer (NSCLC) treatment. After screening of chemotherapeutic agents, tyrosine kinase inhibitors (TKIs) or monoclonal antibody in combination with the putative stem cell killer Salinomycin (SAL), we found Metformin (METF), which modestly exerted a growth inhibitory effect on monolayer cells and alveospheres/CSCs of 5 NSCLC cell lines regardless of their EGFR, KRAS, EML4/ALK and LKB1 status, interacted synergistically with SAL to effectively promote cell death. Inhibition of EGFR (AKT, ERK1/2) and mTOR (p70 s6k) signaling with the combination of METF and SAL can be augmented beyond that achieved using each agent individually. Phospho-kinase assay further suggested the multiple roles of this combination in reducing oncogenic effects of modules, such as ß-catenin, Src family kinases (Src, Lyn, Yes), Chk-2 and FAK. Remarkably, significant reduction of sphere formation was seen under combinatorial treatment in all investigated NSCLC cell lines. In conclusion, METF in combination with SAL could be a promising treatment option for patients with advanced NSCLC irrespective of their EGFR, KRAS, EML4/ALK and LKB1 status.

## INTRODUCTION

Lung cancer is the leading cause of tumor-related death and accounts for the most common malignancy in the world. Epidermal growth factor receptor (EGFR) is highly expressed on the cell surface of > 60% of non-small cell lung cancer (NSCLC) [1]. The ``classic`` EGFR mutations, involving in-frame deletions in exon 19 and the L858R point substitution in exon 21, are representing about 85-90% of all EGFR mutations and associated with dramatic and lasting response to the EGFR tyrosine kinase inhibitors (TKIs) Gefitinib and Erlotinib [2]. However, intrinsic or acquired resistance limits the therapeutic success of these targeted agents. The other EGFR mutations, occur relatively rarely with < 10% of cases, of which the T790M substitution can either be linked to primary resistance to abrogate the inhibitory activity of TKIs, or might be presented as the secondary mutation bypassing the continued requirement for the original target. In general, most instances are associated with acquired resistance [3]. Additionally, the mutation frequencies of KRAS, anaplastic lymphoma kinase (ALK) and liver kinase B1 (LKB1) are approximately 25%, 3-7% and 15-30% of NSCLCs, respectively [4, 5]. Mutations in EGFR, KRAS and ALK are mutually exclusive in individual tumors; however, KRAS oncogene activation is coincident with LKB1 deficiency in 7-10% of all NSCLC [6].

For NSCLC treatment, chemotherapy, molecular-targeted therapy and humanized anti-EGFR blocking monoclonal antibody (mAb) are widely used. Over a decade ago, Metformin (METF), originally developed for type 2 diabetes medication, was shown to decrease cancer incidence and mortality in diabetic patients [7-9]. The ability of METF to lower the circulating insulin level and to stimulate AMPK-mediated suppression of mTOR and protein synthesis may be integral to its anticancer properties [8]. Treated with conventional therapies, tumors may shrink by targeting more differentiated and proliferating cells, whereas typically after 6-10 months, most if not all tumors grow back and become more resistant to the treatment [10]. The cancer stem cell (CSC) model states that a distinct subpopulation of tumor cells with stem cell-like properties is responsible for resistance, metastasis and relapse, leading to the assumption that tumor heterogeneity and hierarchy are generated by aberrant downstream differentiation of one single CSC. Recently, the antibiotic Salinomycin (SAL), acting as a highly selective potassium ionophore and effectively targeting CSCs in several types of cancer, such as breast [11], leukemia [12] and colorectal [13], offers great promise for a more effective systemic therapy. Based on this theory, combinatorial treatment with conventional and CSC specific therapies could provide an effective approach for complete tumor control.

In the present study we showed that METF, an antidiabetic medication with anticancer efficacy, modestly inhibited the growth of NSCLC monolayer cells and their alveospheres/CSCs in a dose-dependent manner, interacting synergistically with SAL. Data indicated the cell growth inhibitory effect of this combination is AMPK independent. Co-administration of METF and SAL further suppressed the EGFR signaling pathway accompanied by inhibition of AKT and ERK1/2 phosphorylation. Remarkably, significant reduction of sphere formation (SF) was seen under combinatorial treatment in all 5 investigated NSCLC cell lines irrespective of their EGFR, KRAS, EML4/ALK and LKB1 status.

## RESULTS

### Characteristics of the NSCLC cell lines used in this study

After chemical genomics based cell line selection, 5 NSCLC cell lines were chosen for evaluation (Table1). Three of them were initially investigated in more detail in order to gain the best combination of drugs that can target both CSCs and differentiated tumor cells. The HCC4006 adenocarcinoma cell line has an EGFR deletion in exon 19 and EGFR amplification with the copy number 5.2, and shows half maximal inhibitory concentration (IC_50_) of Gefitinib at 0.25 μM. NCI-H1975 adenocarcinoma cells, which harbor both the L858R substitution associated with sensitivity to Gefitinib and the “gatekeeper” T790M missense mutation linked with resistance to EGFR-TKIs, are refractory to Gefitinib (IC_50_> 20 μM). The HCC95 cell line is derived from squamous cell carcinoma with wild-type (wt) EGFR and an IC_50_ value of > 10 μM for Gefitinib. Furthermore, all cell lines carry wt KRAS, BRAF and PTEN genotypes; in addition, HCC4006 and NCI-H1975 cells exhibit motility and invasivity (data not shown).

**Table 1:** Characteristics of the NSCLC cell lines used in this study

NSCLC cell line	Histology	EGFR status	KRAS or LKB1 mutation	P53 mutation	IC_50_s of Gefitinib (μM)
HCC4006	AD	del E746–A750, amplification	no	no	0.25
NCI-H1975	AD	L858R and T790M	no	R273H	>20
HCC95	SCC	wild-type	no	no	>20
NCI-H2122	AD	wild-type	KRAS (G12C), LKB1(P281fs^*^6)	Q16L, C176F	>10
NCI-H3122	AD	wild-type	EML4/ALK (E13;A20)	E285V	>10

AD, adenocarcinoma; SCC, squamous cell carcinoma.

**Figure 1:**
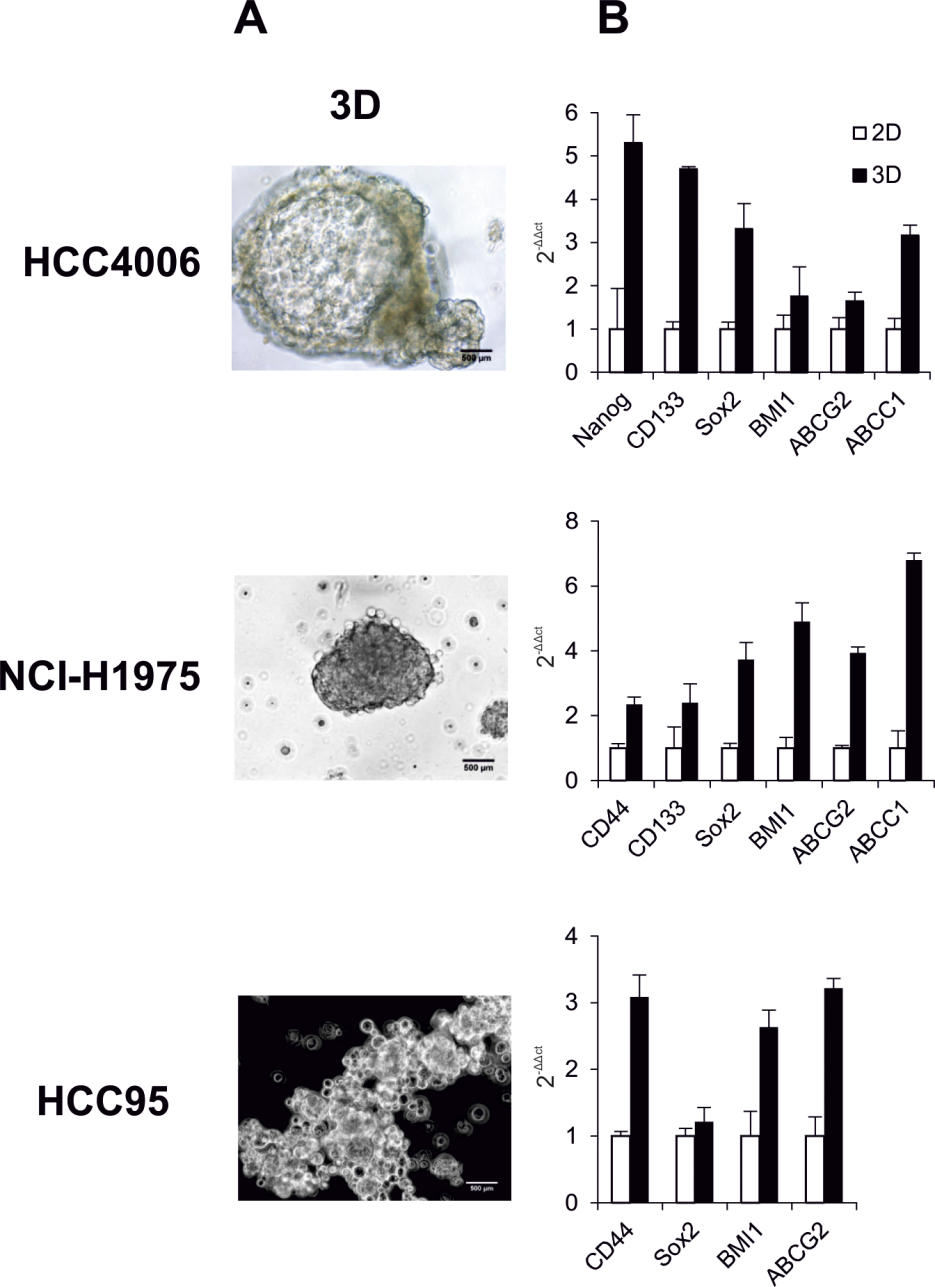
Characteristics of NSCLC cell lines HCC4006, NCI-H1975 and HCC95 (A) Phase-contrast micrographs of alveospheres. Cells were placed in serum-free medium in non-adherent culture flasks to form alveospheres. (B) Real-time PCR analyzed expression levels of stem cell markers and ABC transporters in monolayer cells (2D) and spheres (3D).

Two methodologies were later applied to characterize the CSCs, one of which is SF that is increased with CSC population and function. From the morphology, as shown in Figure 1A, we found HCC4006 cells assembled into highly compact three-dimensional (3D) alveospheres with a spherical cavity inside, whereas the 3D structure of NCI-H1975 cells was less cohesive. In the HCC95 cell line, the initial loose aggregates seen at day 1 gradually and randomly increased into relatively compact spheres after one week in culture. These morphological characteristics of 3D outgrowth of these cell lines could indicate the existence of a functional heterogeneity of CSCs, which may vary from tumor to tumor, depending on their mutations and distinct genetic profiles. Another used strategy is the investigation of stem cell marker (SCM) expression. Until now, to our knowledge, there is no apparent consensus about the “best marker” by which to identify CSCs, hence, here we chose the widely accepted SCMs for characterization. Real-time PCR demonstrated that in HCC4006 cells (Figure 1B, upper panel) the SCMs Nanog, CD133, Sox2, BMI1 and the multi-drug transporters ABCG2, ABCC1 were higher expressed (1.6 ~ 5.2 folds) in alveospheres over two-dimensional (2D) monolayer cells. Similar gene upregulation patterns (2.3 ~ 6.7 folds) were observed in NCI-H1975 3D cells, including additional elevated CD44 expression (Figure 1B, middle panel), whereas HCC95 spheres only showed increased CD44, Sox2, BMI1 and ABCG2 marker expression (1.2 ~ 3.2 folds) (Figure 1B, lower panel). In common, Sox2, BMI1 and ATP-binding cassette (ABC) transporters can be identified as SCMs for the evaluation of CSCs in the tested NSCLC.

### SAL synergizes METF-induced NSCLC cell growth inhibition

**Figure 2:**
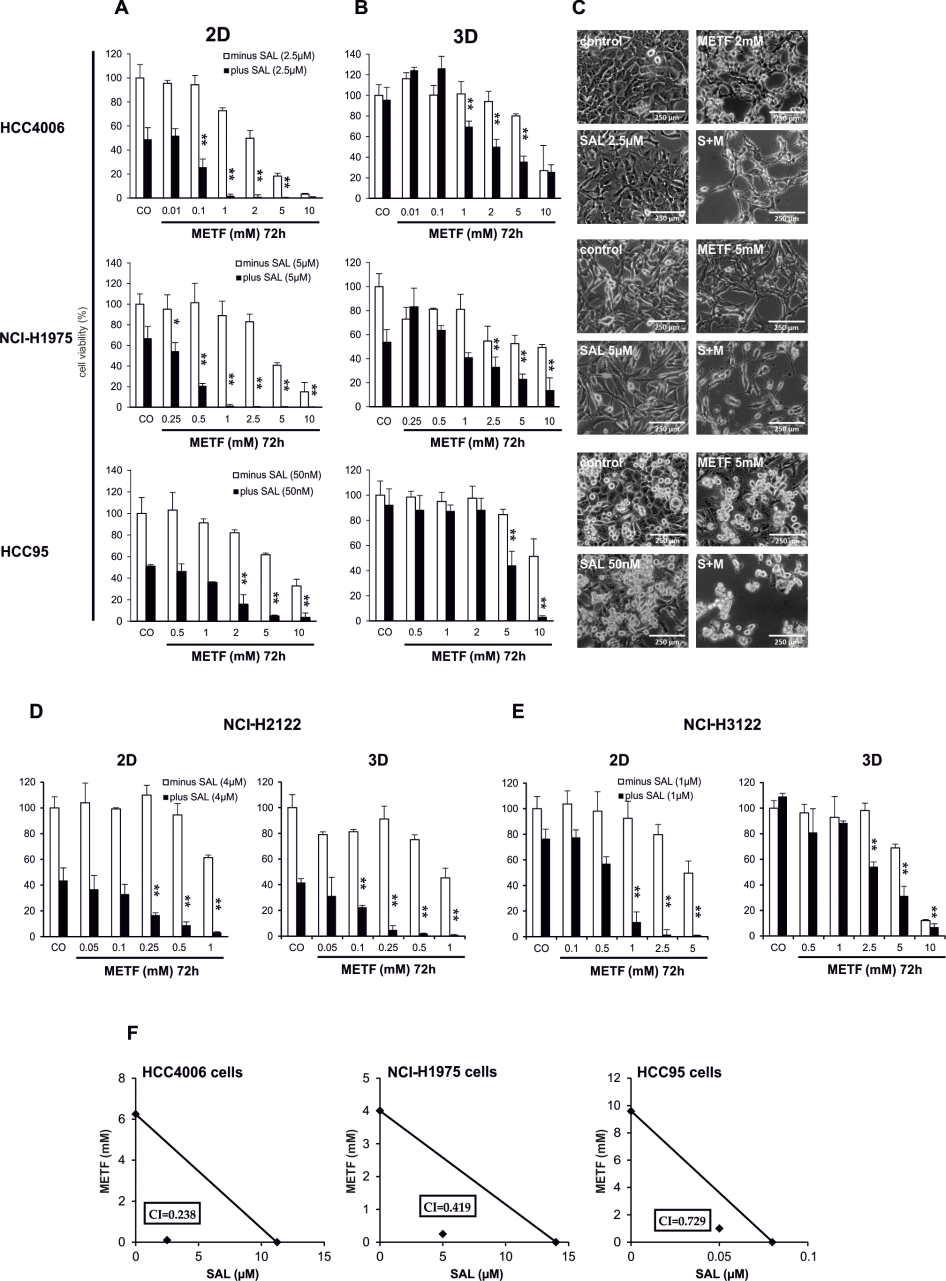
SAL increases METF-mediated effects on cell viability of treated NSCLC Culture HCC4006, NCI-H1975 and HCC95 monolayer cells-2D (A) and spheres-3D (B) for 72hrs in the presence of increasing amounts of METF with and without SAL. Cell growth was assessed using CellTiter-Glo cell viability assay and plotted as a percentage of the viability of DMSO treated cells (control). The asterisks indicate significant differences versus SAL (**P<0.01, *P<0.05). (C) Microscopic examination after 48hrs drug treatment. (D) SAL enhanced growth inhibition after co-administration of METF on extended NCI-H2122 and NCI-H3122 2D and 3D cells. (E) Isobologram analysis of inhibition of cell proliferation with combinatorial treatment. Data points (♦) reflecting the concentrations of METF and SAL were plotted as the ordinate and abscissa respectively, and were represented as average of three independent experiments. CO, control; S+M, combination of SAL and METF.

Out of 12 drugs (Paclitaxel, Carboplatin, Gemcitabine, Gefitinib, Erlotinib, Afatinib, Sunitinib, Dasatinib, Bosutinib, Lapatinib, Erbitux and METF) (Table S1), we found METF modestly inhibited the growth of HCC4006, NCI-H1975 and HCC95 monolayer cells in a dose-dependent manner at the 72hrs time point, as revealed by CellTiter-Glo cell viability assay (Figure 2A). The mean IC_50_ values of METF were 2.5 mM for HCC4006 cells, approximately 5 mM for both NCI-H1975 and HCC95 cells, indicating that the Gefitinib sensitive cell line HCC4006 with the EGFR in-frame deletion displayed the highest sensitivity to METF. Next we examined the growth inhibitory effects of the therapeutic agents in combination with SAL at its IC_50_. Collectively, in all three cell lines, the addition of SAL could not sensitize cancer cells to the 11 therapeutic drugs available in the clinic; however, SAL potentiated the growth inhibitory effects of different concentrations of METF (Figure 2A), as combined treatment caused a significantly greater inhibition in cellular viability relative to either drug alone on 2D cells.

Consistent with growth inhibition, microscopic examination revealed a substantial decrease of cell density and cell death induction upon combinatorial treatment (Figure 2C). More specifically, treatment with 2.5 μM SAL induced vacuole formation in HCC4006 cells, and the number of vacuoles increased after co-administration of METF, whereas either drug alone or in combination promoted NCI-H1975 cells to a more mesenchymal phenotype. HCC95 cells are mainly composed of two different types of cells, which are epithelial-type-cells directly attaching to the plate surface, and apoptotic-like cells sitting on the top of epithelial-like cells, which changed to the mesenchymal type after SAL or METF single application and almost vanished when these two drugs were combined.

Spheres were considered as an *in vitro* model to mimic some aspects of tumor heterogeneity and hierarchy controlled by CSCs. Exposure of alveospheres of HCC4006, NCI-H1975 and HCC95 cells to the same concentrations of METF turned out to be less effective than 2D, whereas co-exposure to SAL significantly enhanced METF efficiency (Figure 2B).

To determine if the cytotoxic effects of this combination are limited to these three cell lines, two additional NSCLC cell lines, namely NCI-H2122 (EGFR wt, KRAS mutation, LKB1 inactivation) and NCI-H3122 (EGFR wt, EML4/ALK translocation), were taken for further investigation. These data confirmed that co-administration of METF and SAL elicited stronger inhibition of 2D and 3D cell growth of these additional cell lines over single treatment (Figure 2D and E). Of note, alveospheres derived from the NCI-H2122 cell line were more sensitive than monolayer cells to either drug alone or their combination (Figure 2D).

To determine whether the combination of METF and SAL has synergistic or merely additive activity, we performed isobologram analysis to assess their inhibitory effects [14, 15]. In our data, specific effects with IC_50_, IC_65_ and IC_75_ levels have been selected for NCI-H1975, HCC95 and HCC4006 cells, respectively (Figure 2F). These 3 data points showed similar cell growth inhibition via co-administration of METF and SAL. As indicated in the isobologram, all dose pairs fell below the straight line, which reflected a synergistic effect. Moreover, treatment of these three lung cancer cell lines with SAL synergized with all indicated concentrations of METF on cell growth inhibition.

Taken together, these findings suggest that METF, which modestly inhibits the growth of NSCLC monolayer cells and alveospheres in a dose-dependent manner, interacts synergistically with SAL.

### The cell growth inhibitory effect of combinatorial treatment with METF and SAL is AMPK independent

**Figure 3:**
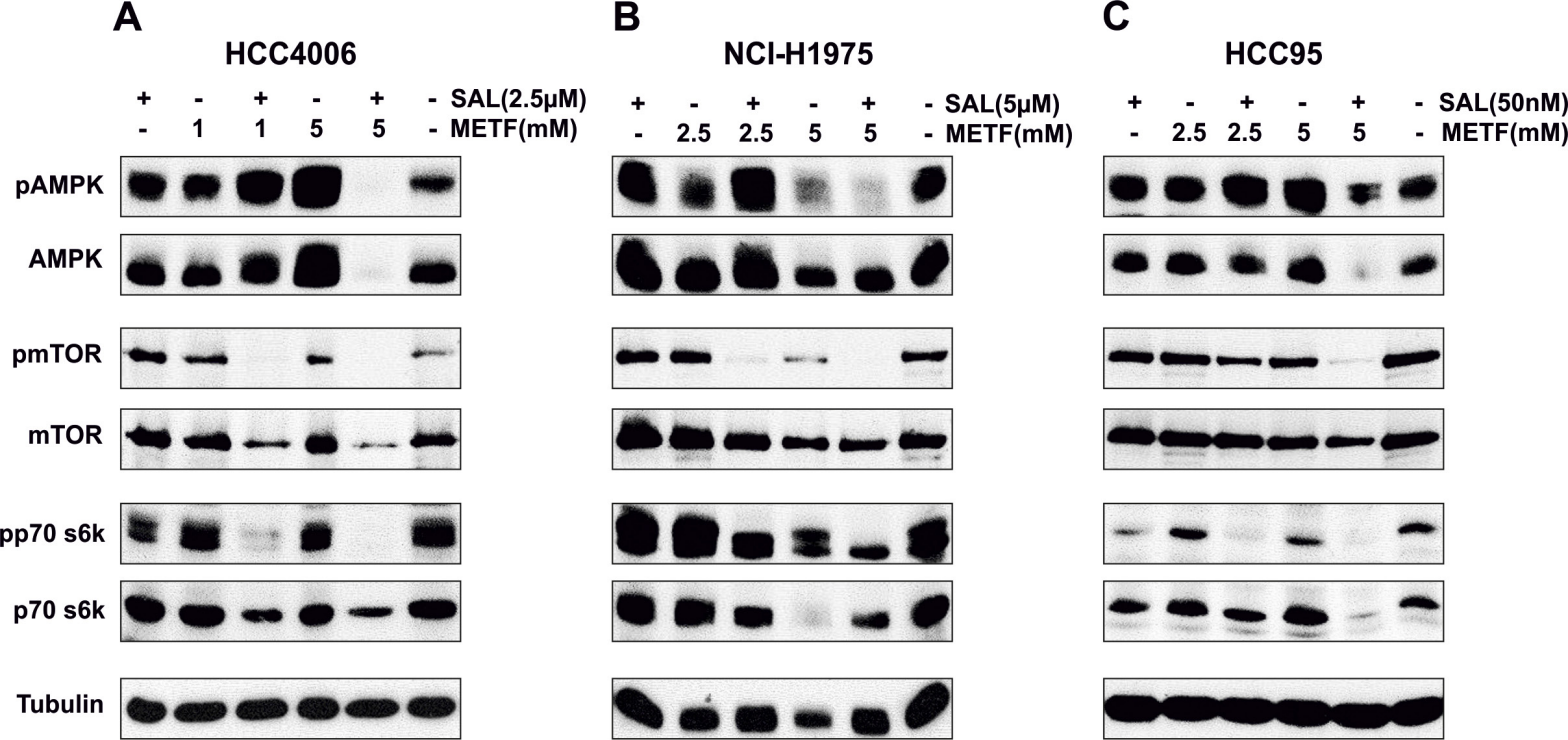
AMPK signaling in NSCLC HCC4006, NCI-H1975 and HCC95 cell lines upon METF and SAL combinatorial treatment (A-C) Monolayer cells were exposed to the indicated concentrations of METF, SAL and their combinations for 48hrs, as specified. After harvesting, cells were lysed and prepared for western blot analysis of downstream molecules of AMPK signaling. Tubulin served as a loading control.

METF, as an AMPK-activating compound, is widely used to suppress cancer cell proliferation. To analyze whether the cell growth inhibitory effect of treatment with METF and SAL is also mediated by activation of the AMPK signaling pathway, several key proteins and associated phosphorylation status have been evaluated. At the indicated two concentrations, METF activated AMPK in a dose-dependent manner in the HCC4006 and HCC95 cell lines (Figure 3A and C), while negatively regulating phosphorylation of AMPK and the downstream molecules mTOR and p70 s6k in NCI-H1975 cells (Figure 3B). These results suggest METF functions as a potent AMPK-independent antiproliferative agent, and AMPK activation may be due to physiological adaptation to metabolic stress. The combination of SAL and lower dose METF (1 mM for HCC4006 cells, 2.5 mM for both NCI-H1975 and HCC95 cells) strongly induced AMPK phosphorylation and associated mTOR and p70 s6k downregulation. In contrast, co-administration of 5 mM METF led to a near-complete abolition of the activated forms of these proteins, and a clear suppression of total protein expression in all three cell lines (Figure 3). Overall, SAL potentiates the inhibitory effect of high dose METF, in our case 5 mM, on NSCLC cell proliferation through unique AMPK-independent mechanisms.

### Characterization of EGFR family signaling in NSCLC cell lines after combinatorial treatment with METF and SAL

**Figure 4:**
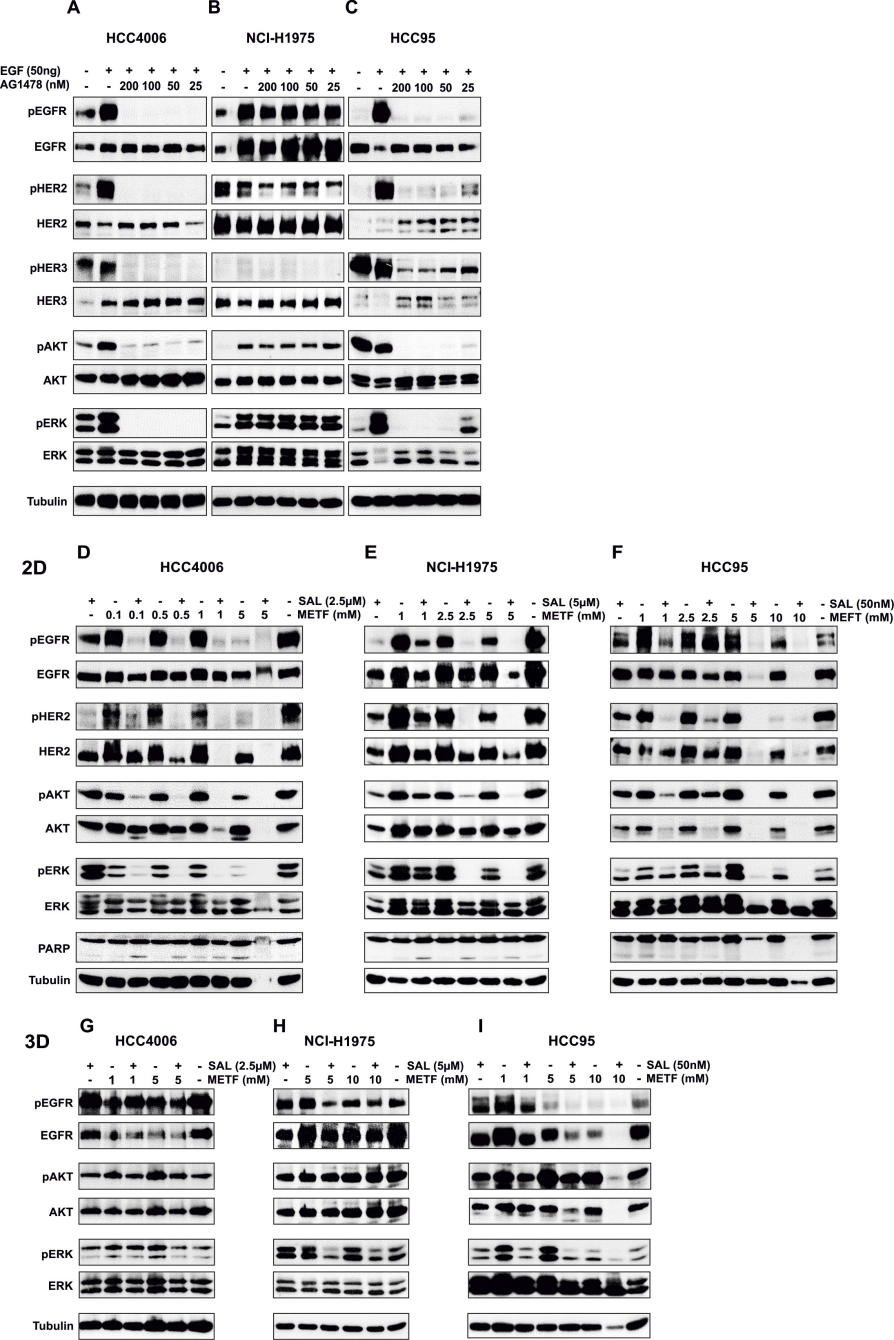
EGFR downstream signaling in NSCLC HCC4006, NCI-H1975 and HCC95 2D and 3D upon METF and SAL combinatorial treatment (A-C) EGFR signaling after EGF and AG1478 treatment. Western blot analysis of extracts from serum starved HCC4006, NCI-H1975 and HCC95 cells, untreated or treated with indicated concentrations of AG1478 TKI for 30 min, followed by stimulation with 50 ng/ml EGF for 10 min. Expression and phosphorylation of EGFR, HER2, HER3, and the downstream signaling molecules AKT and ERK1/2 have been evaluated. (D-F) Monolayer cells-2D and (G-I) spheres-3D were exposed to the indicated concentrations of METF, SAL and their combinations for 48hrs, as specified. After harvesting, cells were lysed and prepared for western blot analysis of downstream molecules of EGFR signaling. Tubulin served as a loading control.

To gain insight into the functional role of the EGFR family in these three pilot cell lines, we examined HER2 and HER3 phosphorylation in serum-starved conditions with and without AG1478 (specific TKI for EGFR) and EGF treatment. After 24hrs serum starvation of HCC4006 and NCI-H1975 cells, EGFR and HER2 were still activated and could be further phosphorylated upon 50 ng/ml EGF stimulation (Figure 4A and B, upper panel). In contrast, there was no EGFR and HER2 phosphorylation in HCC95 cells (EGFR wt), except with the addition of EGF (Figure 4C, upper panel). EGF-mediated EGFR and HER2 phosphorylation was completely prevented by 30 min of AG1478 pretreatment in HCC4006 and HCC95 cells, but persisted at a high level in the NCI-H1975 cell line, as substitution of a threonine at the gatekeeper position (T790M) results in a steric hindrance that may interfere with the binding of TKIs (Figure 4A-C, upper panel) [16]. In HCC4006 and HCC95 cells the preferred heterodimerization partner HER3 was constitutively activated and no further phosphorylation was observed after EGF stimulation. AG1478 treatment entirely inhibited pHER3, as well as pEGFR and pHER2 in HCC4006 cells, whereas the blockade of HER3 transactivation was gradually released along the reduced AG1478 concentrations in HCC95 cells (Figure 4A and C, middle panel). In contrast, under starving conditions, HER3 phosphorylation in NCI-H1975 cells was totally suppressed compared with untreated control cells (data not shown), and remained so despite EGF stimulation (Figure 4B, middle panel).

Concerning the constitutive EGFR phosphorylation in HCC4006 and NCI-H1975 cells, we wished to determine whether the downstream signaling is activated. In HCC4006 cells, EGFR continued to activate PI3K/AKT and MAPK/ERK signaling, and AKT and ERK1/2 could be further phosphorylated with the addition of EGF. AG1478 treatment completely inhibited pERK1/2 and caused a dramatic reduction of pAKT (Figure 4A, lower panel). In contrast, ERK1/2, but not AKT, was constitutively phosphorylated in NCI-H1975 cells (Figure 4B, lower panel). Our findings suggest that the ERK1/2 pathway is preferentially activated by the EGFR T790M mutation. AKT and ERK1/2 can be further phosphorylated with EGF stimulation, but were minimally affected by AG1478 treatment. In HCC95 cells, pAKT is constitutively activated in serum-starved conditions as a result of HER3 permanent phosphorylation, while TKI AG1478 completely abrogated pAKT and pERK1/2 signaling (Figure 4C, lower panel).

These observations suggest that HCC4006 and NCI-H1975 cells, with the somatic gain-of-function mutation and “gatekeeper” T790M substitution, respectively, both undergo EGFR-dependent AKT phosphorylation. However, in EGFR wt HCC95 cells, persistent HER3 signaling is associated with acquired resistance to TKIs by permanent activation of AKT.

To further characterize the downstream EGFR signaling pathway that might correlate with the observed growth inhibition, we examined the effect of the two drugs on the expression of several key regulators acting as biomarkers of response. Using western blot analysis, a weak inhibition of EGFR, HER2 and the downstream regulators of signaling AKT and ERK1/2 protein expression and phosphorylation could be detected after 48hrs treatment of HCC4006 cells with METF or SAL alone, in addition to pHER2 inhibition after exposure to single agent SAL (Figure 4D). In contrast, these endogenous proteins showed a pronounced downregulation after combinatorial treatment. As depicted in Figure 4E, pEGFR was highly sensitive to SAL in NCI-H1975 cells, and the inhibition of pEGFR and pHER2 was METF dose dependent. This combination-induced reduction of phospho-status of EGFR, HER2, and the mediators AKT and ERK1/2 was further augmented after co-administration of 5 mM METF and 5 μM SAL. Cell death development of the tested cell lines was assessed by PARP cleavage. Here we observed that co-administration increased the levels of cleaved PARP in both cell lines (Figure 4D and E, lower panel). In HCC95 cells, single-agent treatment with METF and SAL didn't show obvious inhibition of total and phosphorylated EGFR, HER2, AKT and ERK1/2; however, the activated forms of these proteins were highly suppressed under their combination (Figure 4F).

To corroborate this strong inhibition in an *in vitro* tumor model, multicellular alveospheres were exposed to METF and SAL. Here we mainly focused on the expression and activated forms of EGFR and the downstream mediators AKT and ERK1/2. Clearly, as shown in Figure 4G, alveospheres generated from HCC4006 cells were more resistant than monolayer cells, and a distinct effect was observed at 5 mM METF in combination with 2.5 μM SAL, which displayed in 2D the inhibition of tubulin protein expression. In contrast, NCI-H1975 3D cells can defend higher dose administration, since 5 or 10 mM METF in combination with 5 μM SAL inhibited phosphorylation of EGFR and ERK1/2 compared with single drug treatment, but still not sufficiently to completely block this pathway (Figure 4H). For alveospheres generated from these two cell lines, more rounds of treatment could be necessary for further suppression of proteins mentioned above and induction of cell death. In HCC95 spheres, co-administration of 10 mM METF entirely abrogated pAKT and pERK1/2 (Figure 4I).

### Possible mechanisms of growth inhibition and cell death induction upon combinatorial treatment with METF and SAL

**Figure 5:**
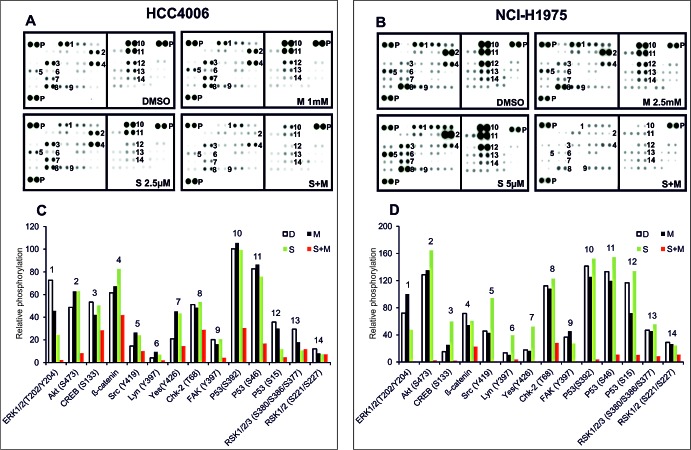
Phospho-kinase array analysis after combined treatment with METF and SAL (A and B) Whole-cell lysates from HCC4006 and NCI-H1975 after treatment with METF, SAL alone or in combination were collected for human phospho-kinase array analysis. Each membrane contains kinase specific (number indicated) and positive control (P) antibodies spotted in duplicate. (C and D) Relative phosphorylation of spots was quantified by normalizing pixel density of the positive control to 100. Each bar is represented as mean of duplicate spots. D, DMSO; M, METF; S, SAL; S+M, combination of SAL and METF.

To get a broader view on signaling inhibition and cell death induction after combinatorial treatment of the HCC4006 and NCI-H1975 cells with METF and SAL, we utilized phospho-kinase array to investigate the phosphorylation of 43 kinases and 2 transcription factors (Figure 5A and B). As mentioned above, these two cell lines represent different EGFR status related to Gefitinib sensitivity. In HCC4006 cells, phosphorylation of ten proteins (ERK1/2, AKT, CREB, ß-catenin, Src, Lyn, Yes, Chk-2, FAK and P53) was downregulated following co-administration of 1 mM METF and 2.5 μM SAL (Figure 5C). The phospho-status of these proteins became more notable when we applied both drugs to NCI-H1975 cells (Figure 5D); besides, ribosomal S6 kinase (rsk) family member phosphorylation also declined upon treatment in combination with 2.5 mM METF and 5 μM SAL (Figure 5D). It is worth to note that in HCC4006 cells, activation of three Src family members did substantially respond to single agent, the same for SAL alone for treatment of NCI-H1975 cells. The finding that combined METF and SAL induced cell growth inhibition, suggests the multiple roles of this combination in reducing oncogenic effects of modules involved in EGFR, Wnt, Src and AMPK signaling, cell adhesion and cell cycle regulation.

### The combination of METF and SAL effectively inhibits sphere formation of NSCLC cell lines with different EGFR, KRAS, EML4/ALK and LKB1 status

**Figure 6:**
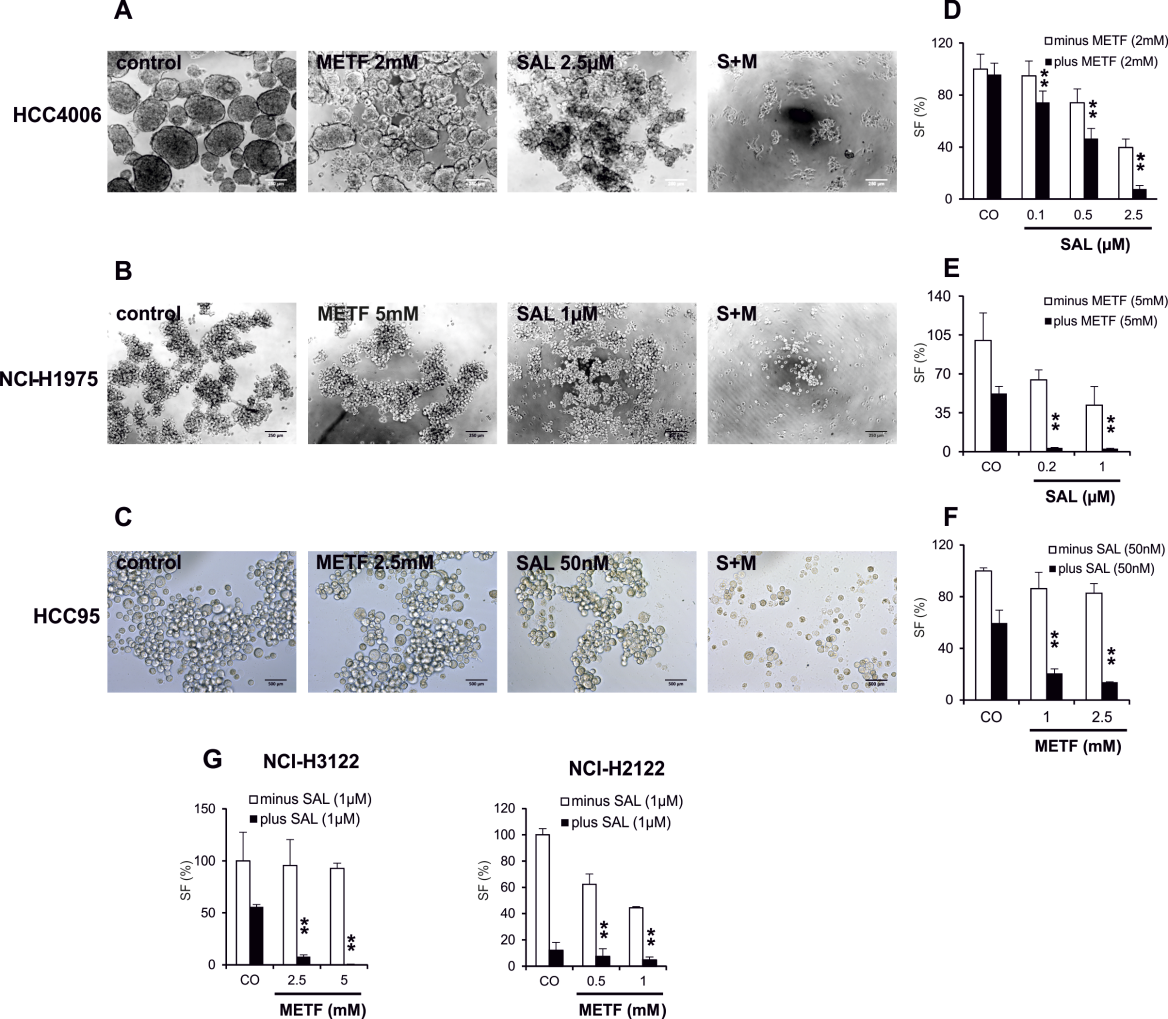
METF and SAL inhibit sphere formation of the NSCLC cells (A-C) Monolayer cells from HCC4006, NCI-H1975 and HCC95 after 48hrs treatment with indicated concentrations of METF and SAL were trypsinized and seeded with 10.000 cells into ultra-low adherent 96-well plate for 4 days of sphere formation with further drug treatment. (D-H) After drug treatment of indicated cell lines, average cell numbers from formed spheres were evaluated by CellTiter-Glo cell viability assay. CO, control; S+M, combination of SAL and METF.

CSCs are characterized by their ability to form spheres after seeding cells in serum-free media [17]. We therefore performed SF assays with cells grown in the presence of METF, SAL, or both. Briefly, monolayer cells were treated with METF, SAL alone or in combination for 48hrs, then trypsinized and replated with 5.000 and 10.000 cells in ultra-low adherent 96-well plates for sphere generation with the continued presence of certain drugs. Representative images of spheres indicated that in HCC4006 cells single agent SAL and, to a lesser extent, METF inhibited SF, whereas their combination prevented generation of spheres most effectively (Figure 6A). At a density of 10.000 cells per well, all combinations especially with 2 mM METF and 2.5 μM SAL showed statistically significant SF inhibition with ±7 folds reduction compared to METF treatment (Figure 6B), and the effect of inhibition was more noticeable when 5.000 cells were seeded (data not shown). Meanwhile, we noted that combinatorial treatment greatly reduced or abolished sphere generation under both amounts of seeded NCI-H1975 cells (Figure 6C and D). Similarly, co-administration of 50 nM SAL with 1 or 2.5 mM METF resulted in 2.5 and 3 folds decrease, respectively, in tumor SF in HCC95 cells (Figure 6E and F).

To confirm these observations, we examined two other Gefitinib resistant NSCLC adenocarcinoma cell lines, NCI-H3122 and NCI-H2122, with the same validation settings (Figure 6G). SF analysis confirmed that combinatorial treatment caused a dramatic reduction in the number of spheres derived from these two cell lines within 96hrs as a consequence of cell death. As an aside, NCI-H2122 spheres exhibited a large reduction of cell viability after SAL single agent treatment; however, co-exposure to METF still enhanced this effect. These data demonstrate the combination of METF and SAL could be a potent killer for lung cancer alveospheres/CSCs.

## DISCUSSION

In the present report we demonstrate the effects of chemotherapeutic drugs, TKIs, mAb, and METF alone as well as in combination with SAL, an agent that could possibly target CSCs, in NSCLC cell lines. The most significant proliferation inhibition and cell death induction activities *in vitro* have been observed upon combinatorial treatment with METF and SAL.

NSCLC causes 1.4 million deaths worldwide every year [18]. Given a wide variety of therapies to which cancer cells are refractory, the CSC theory has brought awareness that eradicating CSCs may overturn the drug-resistance after chemoradiation or targeted therapy. In our study, we did multi-rounds drug treatment on the HCC4006, NCI-H1975 and HCC95 cell lines (with various EGFR status) under combination with the putative stem cell killer SAL and inhibitors for the non-CSC population, such as chemotherapeutic agents, EGFR humanized mAb Erbitux, and a panel of TKIs for EGFR, Src, PDGFR, KIT and VEGFR. The approach of combinatorial treatment with SAL and other inhibitors targeting both CSCs and transit-amplifying/differentiated cells has already been successfully applied to cancers, such as breast [19], pancreas [20] and sarcoma [21]. Unfortunately, SAL didn't enhance the effects of the tested drugs (data not shown), suggesting ABC transporters that are associated with multidrug resistance may not be effectively targeted by SAL in these cell lines. These data are consistent with Larzabal's work [22], which showed differential effects of SAL and Paclitaxel targeting CSC and non-CSC populations, respectively, on lung primary tumors and metastases. However, a combination of both drugs did not improve the effect of single therapies. Collectively, of 12 therapeutic drugs available in the clinic (see Table S1), only METF elicits synergistic effects on the eradication of both differentiated cells and CSCs after co-administration of SAL, thus preventing cancer cell recovery in the tested NSCLC cell lines.

METF is regarded as an anti-hyperglycaemic agent and developed for the treatment of type 2 diabetes. Besides its circulating insulin-lowering effects that reduce fasting blood glucose levels, basic investigations demonstrated that METF also inhibited the growth of various human cancer cell types, such as thyroid [23], prostate [24], gastric [25], breast [26] and glioblastoma [27]. In line with these findings, we for the first time systematically showed that METF exerted a dose-dependent growth inhibitory effects on 5 NSCLC cell lines (HCC4006, NCI-H1975, HCC95, NCI-H2122, NCI-H3122) carrying various EGFR, KRAS, EML4/ALK and LKB1 genotypes irrespective of Gefitinib sensitivity. Furthermore, we found that SAL can sensitize cancer cells to the effects of METF treatment by enhancing inhibition of proliferation and cell death development, validated by CellTiter-Glo viability assay, cell and nuclear abnormalities, and PARP activation (PARP cleavage). Single treatment, such as METF, at the concentrations of 0.07, 0.15, 0.3 and 0.6 mM are without growth inhibition in HCC4006 cells, the same for 0.15 μM SAL. However, in the presence of this combination, we observed a decreasing trend of cell viability that is consistent with METF doses. Here we speculated that, based on its small molecular property, METF, at a concentration less than 1 mM, could be hard to accumulate and can be quickly and effectively pumped out of the cells. Even though it has been proved to have anticancer effect on various cancer cell lines, the published data indicated that the minimum effective concentration of METF is 2 mM. In this case SAL which acts as a possible inhibitor of P-glycoproteins to interfere with ABC transporters [28] preserves the effect of METF even at low dose. Whether they are working synergistically as a complex or individually needs to be further dissected.

Understanding the molecular mechanism underlying the inhibitory activity of METF/SAL is pivotal to develop this combination as a novel therapy to reduce the risk of tumor relapse. EGFR is overexpressed in more than 60% of NSCLC cases and considered as a driving force in lung adenocarcinomas [3], so targeting EGFR to inhibit EGFR-mediated pro-survival and anti-apoptotic signals through the MAPK/ERK and PI3K/AKT pathways would be an effective lung cancer treatment. In HCC4006 cells, EGFR was continually activated in serum-starved conditions and completely abrogated upon EGFR specific TKI AG1478 treatment (Figure 4A). The question arose whether EGFR overexpression was necessary to activate AKT and drive cell survival. We found that knockdown of EGFR using small interfering RNA markedly inhibited HER2 and HER3 phosphorylation, downregulated pAKT and pERK, and increased the levels of cleaved PARP (data not shown). Jeffrey A. Engelman and colleagues [29] showed that MET amplification or the EGFR T790M mutation is associated with Gefitinib resistance through HER3 dependent activation of PI3K/AKT in the presence of TKI. A novel finding of our work is that in NCI-H1975 cells, phosphorylation of AKT is driven by the maintenance of EGFR, as starving conditions sustained the absence of pHER3 (Figure 4B). Our data demonstrate that combinatorial treatment of the NSCLC cell lines with METF and SAL leads to a pronounced “unspecific” inhibition of EGFR, HER2, HER3 and the downstream regulators AKT and ERK1/2 expression and phosphorylation, via yet unknown mechanisms, irrespective of the mutational status and protein expression. Phospho-kinase assay further indicated this combination worked more efficiently on cells harboring the gain-of-function mutant p53 than with wt P53, inducing an almost complete abolition of phospho-serine residues of P53 in Gefitinib resistant NCI-H1975 lung adenocarcinoma cell line. The significance of the P53 mutation for the resistance of this cell line has not been evaluated in our study, however, publicly available data indicated that P53-R273H could increase the resistance to chemotherapeutic drugs and TKIs [30]. Since FAK protein and Src family kinases are involved in cellular adhesion and spreading processes, combinatorial treatment induced the suppression of their activated forms could suggest that the cells are less aggressive after treatment.

There is increasing evidence that 3D spheres more accurately reflect the *in vivo* microenvironment and are suitable as a platform *in vitro* for testing drug delivery, efficacy and CSC involvement. In our current study, cell viability and western blot assays both emphasized alveospheres are more resistant over monolayer cells upon METF alone treatment or co-administration of SAL. As shown in Figure 4G-I, higher doses of both drugs or more rounds of treatment are required to effectively inhibit alveosphere growth as compared to monolayer cells. In particular, an increased concentration of METF in combination with SAL better targeted spheres generated from squamous cell carcinoma HCC95 cells.

In addition to the above-mentioned, SF assays have been widely used to identify stem cells based on their capacity of self-renewal and differentiation *in vitro*. Our present study demonstrate that single treatment with SAL and, to a lesser extent, with METF arrested sphere generation, whereas their combination significantly inhibited SF capability and substantially reduced their size in all tested NSCLC cell lines (shown in Figure 6A-C), reflecting the suppression of self-renewal of CSC.

In conclusion, our data demonstrate that the antidiabetic drug METF synergistically eradicating NSCLC cells in combination with the CSC killer SAL could be a promising treatment option for NSCLC patients irrespective of their EGFR, KRAS, EML4/ALK and LKB1 status.

## MATERIALS AND METHODS

### Cell culture

The human NSCLC cell lines HCC4006, NCI-H1975 and NCI-H2122 were obtained from the American Type Culture Collection (ATCC), and HCC95 and NCI-H3122 were obtained from Dr. Roman Thomas. Cancer cells in monolayer were cultured in RPMI-1640 medium (Gibco) supplemented with 10% FBS (Gibco), L-glutamine, 100 U/ml penicillin and 50 μg/ml streptomycin (Sigma, St Louis). All cell lines were authenticated in 2012 using the StemElite ID System (Promega, Madison, WI).

### Chemicals

Tyrosine kinase inhibitors Gefitinib, Lapatinib, Erlotinib, Bosutinib, Dasatinib and Sunitinib were purchased from Vichem Chemie (Hungary). The therapeutic monoclonal antibody Erbitux was purchased from the Max-Planck Pharmacy in Martinsried (Germany). Chemotherapeutic agents Paclitaxel, Gemcitabine, and putative stem cell killers Metformin and Salinomycin were purchased from Sigma (St Louis, MO). Afatinib was purchased from SelleckChem (Munich), Carboplatin was purchased from Santa Cruze (Santa Cruz, CA), and Tyrphostin AG1478 was purchased from Cell Signaling Technologies (Beverly, MA).

### Sphere formation assay

To examine the effect of drugs in the sphere formation ability, monolayer cells were treated with METF, SAL alone or in combination for 48hrs, then trypsinized and replated with 5.000 and 10.000 cells in ultra-low adherent 96-well plates (Corning, #3474) for sphere generation with the continued presence of certain drugs. Over a 4 day period, cells were transferred into opaque-walled 96-well plates with clear bottoms, and the number of spheres per well was reflected by detection of viable cells. For sphere formation, cells were grown in serum-free culture medium DMEM/F12 (1:1, Gibco) supplemented with 30% Glucose (Sigma G8270), Hepes (Serva 25245), Progesterone (Sigma P8783), Putrescine (Sigma P5780), B27 (Gibco 17504), EGF (Peprotech AF-100-15), FGF (Sigma F0291), ITSS (Roche 110745470), Heparin (Sigma H3149) and NaHCO_3_ (Invitrogen 25080-060).

### Western blot analysis

Monolayer cells or spheres harvested from ultra-low adherent 6-well plates (Corning, #3471) were washed by cold PBS one time, and lysed in lysis buffer containing 50 mM HEPES [pH 7.5], 150 mM NaCl, 1 mM EGTA, 10% Glycerol, 1% Triton X-100, 10 mM Na_4_P_2_O_7_, 1 μg/ml Aprotinin, 1 mM PMSF and 1 mM Orthovanadat. Insoluble cell fragments were removed under centrifugation at 13.000 × g for 20 min at 4°C. 35 μg total proteins together with Laemmli sample buffer were heated at 95°C for 5 min, separated on 7.5 or 10% SDS–PAGE, and transferred to nitrocellulose (Protran BA85, GE Healthcare Life Sciences, US). Membranes were incubated with the primary antibodies diluted in NET-gelatin against pEGFR Y1173 (Cell Signaling Technologies, #4407), EGFR (Transduction Laboratories, E12020), pHER2 Y1248 (Cell signaling Technologies, #2247), HER2 (Millipore, #06-562), pHER3 Y1289 (Cell signaling, #4791), HER3 (Millipore, #05-390), pERK1/2 (Cell Signaling Technologies, #9101), ERK1 K23 (Santa Cruz, sc-94), pAKT (Cell Signaling Technologies, #9271), AKT1/2/3 H-136 (Santa Cruze, sc-8312), pAMPKα (Cell Signaling Technologies, #2531), AMPKα (Cell Signaling Technologies, #2532), pmTOR (Cell Signaling Technologies, #2971), mTOR (Cell Signaling Technologies, #2983), pp70 s6k (Millipore, 07-018), p70 s6k (Millipore, 07-402), PARP (Cell Signaling Technologies, #9542), Tubulin (Sigma-Aldrich, T9026), Secondary HRP-conjugated anti-rabbit (Bio-Rad) and anti-mouse (Sigma) antibodies were used and detection was done using an ECL reagent (PerkinElmer, Rodgau, Germany) on X-ray films (GE Health care Life sciences, US).

### Phospho-kinase array

The Proteome Profiler Phospho-Antibody Array Kit (ARY003, R&D Systems, US) was used following manufacturer's instructions. Spot densities were analyzed using the AIDA software (Raytest, Germany). The average density of duplicated spots was determined and normalized for the relative changes of phosphorylation of 43 kinases and 2 related total proteins.

### Proliferation assays and isobologram analysis

Opaque-walled 96-well plates with clear bottoms (Corning, #3603) and ultra-low adherent 96-well plates were used for 2D and 3D culture, respectively. The number of cells used per cell line was determined empirically, cells from HCC4006, NCI-H1975, HCC95, NCI-H2122, NCI-H3122 were seeded at 5 × 10^4^, 7.5 × 10^4^, 1.3 × 10^5^, 2.2 × 10^5^, 1.2 × 10^5^ cells/well, respectively. Drug treatments were performed next day after seeding and proliferation was measured 72hrs later using CellTiter-Glo luminescent cell viability assay (Promega), following manufacturer's instructions. As a slight adaptation to sphere specificity, the mixture was transferred into an opaque white cell culture 96-well plate after pipetting up and down 3 times, and incubation time was extended to 20 min accordingly. Each point represents a percentage of the DMSO treated control. Experiments were set up in 4 replicate wells and repeated thrice. According to the Chou-Talalay method for evaluation of drug combination, the combination index (CI) was calculated using the CalcuSyn software (Biosoft) and quantitatively defined the effect between two drugs, with CI < 1 (synergism), CI = 1 (additivity) and CI > 1(antagonism).

### Real-time PCR

RNA was extracted using the RNAeasy kit (QIAGEN) and 5 μg isolated total RNA was reverse-transcribed into cDNA as template for PCR amplifications using AMV Reverse Transcriptase (Roche Diagnostics, Mannheim, Germany). All quantitative PCR reactions (20 μl) were carried out in the StepOne plus Real-Time PCR system (Applied Biosystems) using Fast SYBR Green Master Mix (AB Applied Biosystems, Darmstadt, Germany). The 2 ^−ΔΔCT^ method was used to analyze the relative fold change in gene expression with hypoxanthine phosphoribosyltransferase (HPRT) (Applied Biosystems) as an endogenous control. All specimens were evaluated in triplicates. Details of the primer sequences are in Table S2.

### Statistical analysis

Data were represented as mean ±SD from three independent experiments unless stated otherwise. Statistical analysis was performed by one-way analysis of variance (ANOVA), and statistical significance (**P<0.01, *P<0.05) was evaluated with the unpaired 2-tailed Student t test to assess difference between treated and control samples. All data were done using R 2.13.2 (R Foundation for Statistical Computing, Vienna, Austria).

## SUPPLEMENTARY TABLE


