# Supplementary material for: Metformin and salinomycin as the best combination for the eradication of NSCLC monolayer cells and their alveospheres (cancer stem cells) irrespective of EGFR, KRAS, EML4/ALK and LKB1 status
Source: Oncotarget. 2014 Nov 2;5(24):12877–90. doi: 10.18632/oncotarget.2657 (PMC4350329; doi:10.18632/oncotarget.2657)
Supplement: Supplementary file 1 [file oncotarget-05-12877-s001.pdf]

Metformin and salinomycin as the best combination for the eradication of NSCLC monolayer cells and their alveospheres (cancer stem cells) irrespective of EGFR, KRAS, EML4/ALK and LKB1 status

Supplementary Material

Table S1. Single-drug effects on HCC4006, NCI-H1975 and HCC95

IC_{50} (μM) / Drug \ Cell line	HCC4006	NCI-H1975	HCC95
Paclitaxel	0.1	1.4	>20
Carboplatin	130 μg/ml	52.3 μg/ml	63.5 μg/ml
Gemcitabine	>10	5.8	>20
Sunitinib	>10	>20	>20
Afatinib	0.0082	1.3	16.3
Erlotinib	0.15	>10	>20
Gefitinib	0.25	>20	>20
Lapatinib	3.78	>10	>20
Dasatinib	3	>10	20.3
Bosutinib	7.3	12.8	16.8
Erbitux	>200 μg/ml	>200 μg/ml	>200 μg/ml
Metformin	2.5 mM	4 mM	6.8 mM
Salinomycin	2.5	5	0.05

Table S2. Primers used for real-time PCR [31-36]

Primers	Sequence (5´-3´)
ABCC1-F	ATGTCACGTGGAATACCAGC
ABCC1-R	GAAGACTGAACTCCCTTCCT
ABCG2-F	AGATGGGTTTCCAAGCGTTCAT
ABCG2-R	CCAGTCCCAGTACGACTGTGACA
BMI1-F	GATGAATTCGTCACTGTGAATAACGATTT
BMI1-R	TCTAGATCTACAATCATTTCTGAATGCAT
CD44-F	TCCAACACCTCCCAGTATGACA
CD44-R	GGCAGGTCTGTGACTGATGTACA
CD133-F	GCTCAGACTGGTAAATCCCC
CD133-R	GACTCGTTGCTGGTGAATTG
Nanog-F	CAAAGGCAAACAACCCACTT
Nanog-R	TCTGCTGGAGGCTGAGGTAT
Sox2-F	TCCCATCACCCACAGCAAATGA
Sox2-R	TTTCTTGTCGGCATCGCGGTTT
hHPRT-F	TGACACTGGCAAAACAATGCA
hHPRT-R	GGTCCTTTTCACCAGCAAGCT
